# Indigenous Infant Mortality by Age and Season of Birth, 1800–1899: Did Season of Birth Affect Children’s Chances for Survival?

**DOI:** 10.3390/ijerph15010018

**Published:** 2017-12-23

**Authors:** Lena Karlsson

**Affiliations:** Centre for Demographic and Ageing Research, Umeå University, Umeå 90187, Sweden; lena.karlsson@umu.se

**Keywords:** indigenous, infant mortality, season of birth, Sami, Sweden

## Abstract

This paper focuses on the influence of season of birth on infant mortality among the Sami and non-Sami populations in northern Sweden during the nineteenth century. The source material is a set of data files from the Demographic Data Base at Umeå University, making it possible to combine age at death (in days), month of death, and month of birth over the course of the entire century. Cox regression models reveal that for the first week of life, season of birth had no influence on the risk of mortality. For the Sami, the results showed that being born during winter was related to a higher risk of neonatal mortality, and being born during summer was related to a higher risk of mortality after six months of age. Furthermore, for the Sami, the neonatal mortality showed a U-shaped pattern with a minimum in June–August, whereas the corresponding pattern among the non-Sami was flatter. The findings shed light on vulnerability in two populations sharing the same environment, but diverging in terms of social, economic, and cultural factors.

## 1. Introduction

From previous research about the demographic transition in Swedish Sápmi (the Sami’s traditional lands), we know that infant and child mortality were generally higher among the Sami compared to the non-Sami population living in the same area [1,2,3]. For example, during the period of 1800–1849, the infant mortality rate among the Sami in Jukkasjärvi was almost twice as high compared to the non-Sami [1]. Previous research has also revealed large differences in infant mortality between regions in Sápmi during the nineteenth century, for example a generally higher infant mortality rate in Jukkasjärvi compared to the corresponding rate in Jokkmokk [1].

In historical populations, the risk of infant mortality has been shown to vary by season or month of birth [4,5]. While previous research has revealed an unequal distribution of infant mortality among the Sami and non-Sami populations, there are only a few studies covering the effect of season of birth on overall infant mortality among the Sami population. Sköld et al. found no seasonal differences in infant mortality during the nineteenth century among the Sami, except in Föllinge parish, where higher infant mortality rates appeared among infants born during the winter months [1]. Brändström found in his study that neonatal mortality among the Sami population in Jokkmokk was highest during the summer months (July and August), followed by the winter months (January through March) [6]. Compared to the Sami population, the neonatal mortality among the non-Sami population did not show any significant seasonal variations [6].

Previous research concerned with season of birth and infant mortality during the nineteenth century has shown two major patterns, where mortality was higher among infants born in the winter (Italy, Belgium, and Spain) or higher among infants born in the summer (Russia and Poland). The first pattern has been explained as a combination of cold climate and a high risk of respiratory infections, where newborns are at a higher risk of dying of respiratory diseases during the second and third weeks of life [4,7,8]. For example, in Italy, an infant born during winter was almost four times as likely to die compared to infants born in summer [7]. Furthermore, northern and central Italy [4,9] showed a U-profile of the neonatal mortality by month (or season of birth), which was mainly explained by harsh winter conditions. The second pattern has been explained as a combination of hot climate and higher risk of contracting infections of the digestive tract [4,5]. The higher neonatal mortality found among Russian infants born in summer also relates to social and cultural factors, where mothers were expected to participate in agriculture and thus leave the home and take less care of the infant [4,5]. So far, however, the evidence to support both explanations is inconclusive. Thus, both climatic and cultural determinants might contribute to differences in seasonality.

Further, the association between season of birth and the risk of death is also related to the age of the infant [5]. Generally, the infant mortality rate decreases with each succeeding month of age. This decreasing pattern seems to be interrupted when the summer-born infant encounters the harsh conditions of winter [10] or when the winter-born infant enters the summer season [11]. The month or season of birth also determines the age at which a child encounters seasonal epidemics. For example, infants born in summer are about 6 months old when they enter the winter season (when respiratory epidemics are widespread) and are also at the age when dietary patterns are often changed through the introduction of solid food and less breastfeeding. Around that age, infants also lose their natural protection obtained from their mother that provided immunity against certain diseases.

Breschi and Livi-Bacci [4] related season of birth, age, and mortality to factors such as breastfeeding practices and the incidence of infections. For instance, among children born in the winter, the risk of respiratory infections was high during the first days and weeks of life, which could be reduced by adequate protections (heating, less exposure to infections, etc.). Among children born in the spring–summer, the risk of contracting infections of the digestive tracts was higher, especially among non-breastfed infants. Children born in summer and weaned before their first birthday were found to have a higher mortality risk during their second summer compared to those children still being breastfed [4].

### Seasonality, Nursing Practices, and Living Conditions in Sápmi

The distribution of infant mortality by season of birth gives important information about possible components behind infant mortality rates [6]. By studying seasonality, we can relate infant mortality to cultural and social aspects to a greater extent, for example, to the varying work intensity of reindeer-herding Sami during the year, to housing/living conditions, delivery of infants, and breastfeeding practices [1,12]. In this northern rural area, delivery of infants was still a private matter, taking place in one’s home [13]. Proportionally more Sami than non-Sami children were born during the winter season during the nineteenth century [6]. In Sápmi, Sköld found that the general mortality (in the total population) was highest during the winter months (December–Feburary) and June [14]. While the seasonality of births has been related to religious, social, and economic factors, the seasonality of deaths has to a greater extent been associated with climate-related factors [11]. Regarding the season of death in Skellefteå in northern Sweden between 1749 and 1859, Rocklöv et al. found that colder winters did not lead to higher death rates among infants, whereas they did have an effect on children between three and nine years of age [15].

During the nineteenth century, breastfeeding practices diverged in Sweden, and in many places it was not common to breastfeed infants. In order to decrease the high infant mortality rate in Sweden several interventions were arranged, such as breast-feeding and child rearing campaigns [1,6]. Compared to the general trend, breastfeeding was the only option for Sami mothers, and the infant nursing practices among the Sami population were often used as a good example. According to clergy, physicians, and travellers, the Sami children were breastfed for 2–4 years [1,16].

The reindeer-herding year was traditionally divided into eight different seasons that illustrated the shifting reindeer-herding conditions and work intensity. For the Sami, this traditional nomadic lifestyle played an important part in the high infant mortality, and Sami women only had a couple of days after giving birth before moving on the next settlement [17,18]. During the stay in the summer settlement, from the end of June and a couple of weeks forward, the work load was intense for the reindeer-herding Sami, with the labelling of calves that often took place during night-time. Compared to the Sami men, Amft found that during this period (June–August), the Sami women with infants or who were pregnant had the least work-intensive period [19].

The shortcomings in previous research accounting for the impact of season of birth on infant mortality have mainly been due to limitations in the available data. The data used in this study made it possible to combine all three parameters—age at death (in days), month of death, and month of birth—among the Sami and non-Sami populations living in Sápmi during the entire period of 1800–1899.

This study analysed differences in mortality according to season of birth between the Sami and non-Sami populations in two parishes (Jokkmokk and Jukkasjärvi) during the nineteenth century according to (1) the overall infant mortality, (2) the neonatal mortality, and (3) age profiles of deaths during the first year of life. 

## 2. Materials and Methods

### 2.1. Demographic Data

The source material for this study was a set of data files from the Demographic Data Base at Umeå University [20,21]. Since 2002, parish records from the Sápmi area from the eighteenth and nineteenth centuries have been digitized and linked by the Demographic Data Base [21]. The dataset is a result of a combination of sources, including records of births, deaths, marriages, and migration, as well as catechetical examination registers. The different records and sources belonging to the same individual in the parish are linked so that every individual who lived in the parishes is included and can be followed from birth to death [21]. This is possible because the clergy regularly updated the register [22]. The database contains all individuals born in, or migrating into, the parishes. The data has some shortcomings, since delivery of infants was a private matter in one´s home, data lacks information about vital factors associated with infant’s health and risk of mortality, such as low birth weight, congenital defects, and prematurity [23,24]. Although there are certain shortcomings involved with studying historical materials, there are many advantages as well. The data are unique because they allow the study of mortality patterns in Sápmi on a comprehensive scale and over an extended period of time. Furthermore, they distinguish between the Sami and non-Sami groups (see Variables). The data for this study provide a unique opportunity to study infant mortality according to season of birth within this specific cultural context, where the Sami lived as the majority prior to the onset of colonisation and the mass migration of non-Sami into the region. 

#### Variables

Only infants born within the study area and with a known date of birth and date of death have been included when calculating infant mortality rates. In the data, children of unknown birth or death were given a generic birthday of the 1st of January or a generic date of death of the 31st of December. Because the aim of this paper was to study infant mortality according to season of birth (month), those individuals identified with generic dates of birth and/or death were treated as missing cases. By this we avoid overestimating deaths during winter (December) and underestimating infant mortality among those infants born in January. Infant mortality rates were calculated as the number of deaths within the first year of life per 1000 live births during a time period (i.e., stillbirths are excluded). Neonatal mortality rates were defined as deaths in the first 28 days of life [25].

Ethnicity was not registered in the church records, but the database provides opportunities to find information about a person’s ethnicity. The material distinguishes the indigenous Sami population from the non-Sami population through a system of ethnic indicators designed and implemented by historian Gabriella Nordin [26]. The sources used to find indications of ethnicity are occupation, mortality records, geographical information, name, and family relations. Inclusion of the word “Lapp”, “Lappish”, or “Nomad” is the most prominent indicator of Sami ethnicity found in the different sources [22,26]. The non-Sami group includes the settlers who began moving to the Sami area in the mid-eighteenth century, mostly from the northern coast of Sweden and Finland [1,26].

When analysing if season of birth affected the infant’s chances of surviving the first month and year of life, the months were categorized into season of birth as Winter: December–February, Spring: March–May, Summer: June–August, and Autumn: September—November. 

### 2.2 Methods

The risk of infant mortality was modelled as a Cox proportional hazard model with season of birth, sex, parity, and region as explanatory variables. Separate models were estimated for infant mortality according to age at death during the first year.

This study expected to find differences in infant mortality between different stages during the first year of life according to the standard of living and climate condition in this subarctic area of Sweden and differences between the Sami and the non-Sami populations according to different cultural practices. In order to separate genetic and familial factors important at the very beginning after birth from those that are important at later stages (including economic, social, environmental and cultural factors), the categorizations of 0–7, 8–28, 29–179, and 180–365 days after birth were used. Child mortality (death at ages 1–4 years) was included to see whether the impact of season of birth had a lingering effect on mortality [24]. 

## 3. Results

### 3.1. Seasonal Births and Mortality in Sápmi

In order to analyse the impact of season of birth on infant mortality, it is important to take into consideration the seasonality of the two other vital events, births and deaths. In Table 1, the births and deaths are presented by ethnicity and month of birth. Generally, for both populations, births and deaths were over-represented during the first three months (January–March). 

**Figure 1:**
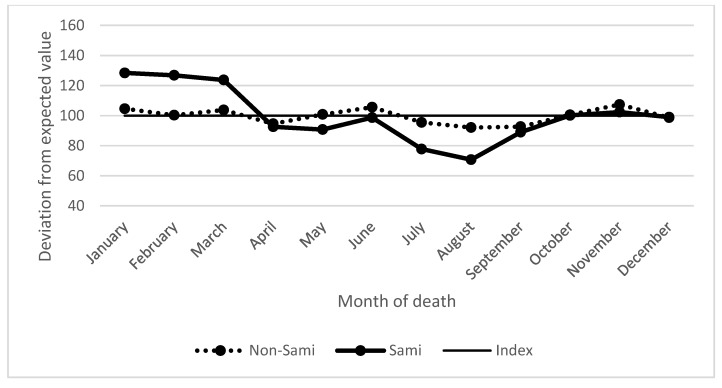
Risk of death by calendar month shown as the deviation from the expected value (100). Sami and non-Sami infants in North Sápmi. Source: Demographic Data Base, Umeå University.

Figure 1 presents the number of events (deaths) in each of the 12 months expressed as index numbers, where 100 represents the expected proportion of deaths after taking into account the number of days in each month, and each deviation is expressed as the percentage above or below the expected proportion. The seasonality of deaths among Sami infants shows a strong peak during the months of January (28% higher than expected), February (27% higher than expected), and March (24% higher than expected), and a decreased pattern during the spring, summer, and early autumn, and this pattern is close to the inverse of the temperature curve (Figure 1). Contrary to the Sami, the mortality pattern among the non-Sami infants was more flat with a tendency to lower mortality during summer (July and August) (Figure 1). 

### 3.2. Mortality Rates by Month of Birth

**Figure 2:**
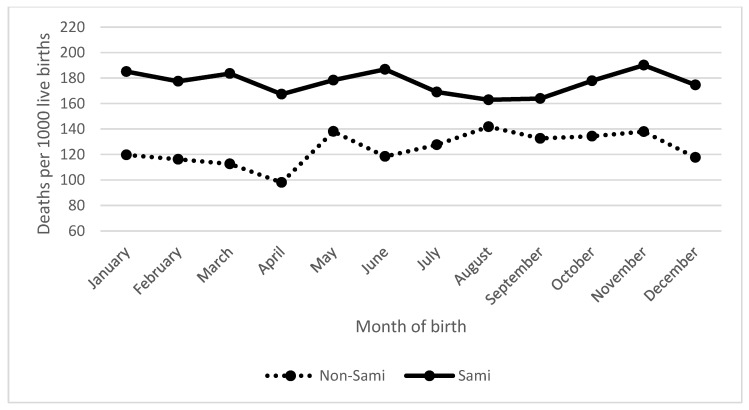
Monthly variation in infant mortality rate (q0–11) by month of birth, Sami and non-Sami populations, 1800–1899. Source: Demographic Data Base, Umeå University.

Because the samples were too small to include separate analyses for different periods of the nineteenth century, the following analyses used the entire time period (1800–1899). Figure 2 presents infant mortality rates (q0–11) among the Sami and non-Sami populations by month of birth.

The Sami population reveals differences in mortality according to season of birth, where children born in November had the highest infant mortality rate, 190 deaths per 1000 live births, compared to 163 per 1000 among children born in August (Figure 2). In more general terms, infants born in late summer and early autumn (July–September) were at a more advantaged position. Contrary to the Sami population, non-Sami children born during the summer (in August) had the highest infant mortality rate (142/1000), and the lowest rate was among non-Sami infants born in April (98/1000) (Figure 2). The non-Sami infants born in winter months (December, January, and February) or spring months (March and April) had generally lower mortality rates compared to non-Sami infants born in summer or autumn.

### 3.3. Neonatal Mortality by Month of Birth

**Figure 3:**
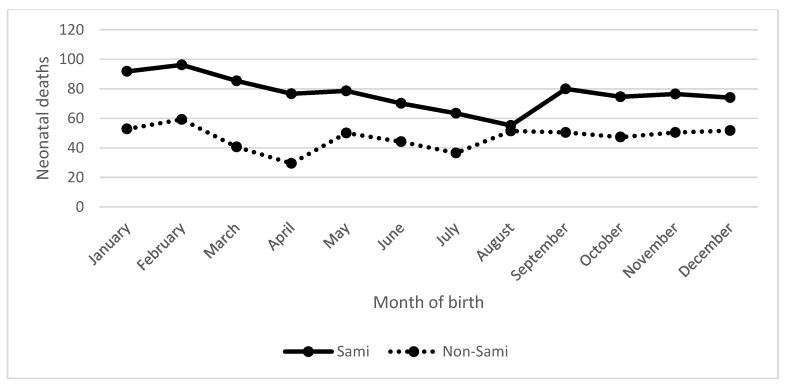
Neonatal mortality (q0–1) in North Sápmi by month of birth, 1800–1899. Source: Demographic Data Base, Umeå University.

The neonatal mortality by month of birth is compared between the Sami and the non-Sami populations in Figure 3. The neonatal mortality rate was highest among Sami infants born in February and lowest among infants born in August, at 96/1000 and 55/1000 live births, respectively. Similarly as for the Sami, the non-Sami infants born in February had a higher risk of dying during the first month of life (Figure 3) and a decreased risk during April, at 59/1000 and 29/1000 live births, respectively. Compared to the Sami, August was one of the months where the neonatal mortality was highest (51/1000 live births). Generally, the non-Sami neonatal mortality revealed a flatter pattern compared to the Sami.

### 3.4. A Model of Season of Birth and Age at Death

This section combines the infant mortality by age and season of birth. Because of the small numbers, the months were grouped as seasons—winter, spring, summer, and autumn (see materials and methods section). As presented in the introduction, this study hypothesized that the influence of season of birth on the mortality risk differs according the different stages an infant goes through during the first year of life. At some points in time, the season of birth is relatively unimportant, and at other points in time it plays a more central role, and the direction might change during the first year of life, with a protective season of birth becoming a dangerous season and vice versa [4,10,11].

During the first week of life, this study expects the risk factor “constitution at birth” and endogenous factors, here measured by sex and parity, to be vital for infant survival. According to previous studies, boys are at a higher risk of death during the very beginning of life [24]. Regarding parity, studies have shown a J-shaped curve, where the mortality risk decreases after the first child and then again increases for four or more children [27,28]. This higher vulnerability among first-born infants is especially clear for neonatal mortality [23]. At this stage, as found in previous studies, infant mortality should be higher among boys [29] and among firstborns due to difficulties associated with the first delivery [30]. At this stage, we expect no influence of season of birth on mortality risk.

During the second period (8–28 days), the risks of mortality are expected to be higher among infants born during the winter period [4]. Following previous research and the mortality patterns described above, we hypothesized that winter-born Sami infants had the greatest risk of neonatal mortality, whereas infants born in summer experienced the lowest levels of neonatal mortality. The cold and harsh climate during winter in combination with movements between settlements are likely to be factors related to an excess in neonatal mortality among the Sami. Among the non-Sami infants, the summer-born infants are hypothesized to be at a higher risk due to women’s participation in agriculture, which means less breastfeeding and higher risks of food contamination. 

After the first month (29–179 days), previous research has revealed that poor conditions, starvation, etc. (related to environmental, social, and economic factors), have a greater impact on mortality [31]. Sami infants born during spring and (early) summer should still be at an advantaged position, whereas infants born during autumn should be at a higher risk when entering the first winter. At this stage in life, being born during autumn is also hypothesized to be associated with a risk among the non-Sami infants.

For the fourth period (180–365 days), the early protective influence of being born during summer is here expected to become a disadvantaged season of birth when entering the first winter at about six months of age, seen as higher mortality risks especially among the Sami infants.

In Table 2, the frequencies (deaths) and mortality rates among the Sami and non-Sami populations reveal a somewhat different pattern, where the mortality rates were higher among Sami infants aged 8–28 days compared to newborns (0–7 days old), and the opposite pattern was found among the non-Sami. Further, the differences in rates between the two populations were largest from the age of 8 days to 28 days after birth, whereas the difference was smallest during the period of 180–365 days (34/1,000 and 29/1000 live births, respectively). In order to perform Cox regression where the assumption that the effects of the covariates do not vary with age, each age group was analysed in a separate model.

For the first week of life (0–7 days), the season of birth had no relationship with the risk of death (Table 3). As expected, the birth order influences the risk of infant mortality, where the firstborn were at a disadvantaged position (there was only a significant increase among the non-Sami), and there was a higher risk among Sami and non-Sami boys. Continuing with the risks of mortality between 8 and 28 days, Sami infants born during summer had a 0.6 times lower risk of dying compared to infants born during winter, whereas the non-Sami show a similar but nonsignificant pattern. Interestingly, for the period of 29–179 days, the season of birth revealed no impact on the risk of mortality. Among the non-Sami, a higher-order parity was associated with a lower mortality risk, and among the Sami there was a higher risk of mortality among boys. At the age between 180 and 365 days, the impact of being born during summer changed direction from being protective to becoming dangerous. The risk of mortality among Sami infants born during summer were 1.7 times higher compared to infants born during winter, and the corresponding risk among non-Sami infants was 2.3 times higher.

Season of birth still had an impact on mortality among the non-Sami children 1–4 years old, where the risk of death was highest among children born during autumn (1.3 times higher, Table 3). Interestingly, among the Sami, parity changed direction, where a higher number parity (four children and above) increased the risk of mortality. 

As shown in this section, the season of birth made a significant difference in terms of surviving at the age of 8–28 days (Sami) and at the age of 180–365 days. Among the non-Sami population, this importance of season of birth was not restricted to infancy and lasted into the ages of 1–4 years. 

## 4. Discussion

The present study has revealed how season of birth affected infant mortality among two populations that differed in terms of cultural, social, and economical aspects but shared the same environmental conditions. The results revealed an excessed infant mortality among the Sami during the winter months, a mortality pattern similar to what was found in other parts of Sápmi and Sweden as a whole during the nineteenth century [14,32]. The results also showed that the risk of death (<1 year of age) among the Sami was highest among infants born during the cold winter months and lowest among those born during the summer months of June to August. For the non-Sami, the infant mortality generally was highest among infants born during the autumn (August to November) and lowest among those born during the spring (April). Late summer/early autumn corresponds to the peak of work in agriculture, and the participation of women during these times risks the health of the infant [29]. In previous research, a higher rate of female work participation has been related to earlier weaning and to irregular breastfeeding of children [4]. In the non-Sami population, the agricultural work might have prevented the mothers from breastfeeding their infants causing higher infant mortality among infants born in August.

The neonatal mortality rates among the Sami and non-Sami were both higher during the winter season, with a peak among infants born in February. Among the Sami, this was a period when conditions both outdoors (extreme cold) and inside the hut (with wood smoke from open fires) were more problematic [1]. Following the working cycle of the reindeer-herding Sami, February corresponds to the period at the winter settlement, whereas June–August (with the lowest neonatal mortality) corresponds to the less work-intensive period, especially for women, in the summer settlement. While the neonatal mortality rate dropped to the lowest level among the Sami in August, it was the month with the second highest mortality rate among the non-Sami, which corresponds to a work-intensive period for non-Sami women. In Sweden, August is recognized as the “dog days” with higher risks for food positioning, contaminated water, etc. [33]. This warm period, when cow milk went sour and infections flourished, in combination with a higher work load among the women (and thus less breastfeeding), might explain the more severe period for the non-Sami infants compared to their Sami counterparts. This difference between the two populations supports the hypothesis that infant mortality in historical populations depends upon mother’s participation in labour during infancy (less breastfeeding, higher risk of contaminated cow milk, etc.). This lower risk of neonatal mortality during summer among the Sami was significant even when controlling for other demographic factors.

The results presented in this article are both consistent and inconsistent with previous findings regarding infant mortality in historical populations. As found in the studies of Breschi and Livi-Bacci [4], the neonatal mortality was highest among winter-born Sami and lowest among infants born during summer. Several studies on infant mortality in northern and central Italy [4,9] showed U-profiles of neonatal mortality by month (or season of birth), and these were explained as a relationship with low winter temperatures [8,27]. A similar pattern was also found among the Sami living in North Sápmi, and contrary to Brändström [6] August had the lowest neonatal mortality. In contrast to studies from the Mediterranean region, the harsh winters with extremely low temperatures in this arctic area of Sápmi constitute a dangerous season with regards to neonatal mortality and for summer-born infants entering their first winter.

The results also indicate a different disease panorama between northern Sweden and countries in the Mediterranean region where previous research on the association between season of birth and infant mortality among historical populations has been conducted [4,11]. While respiratory infections are more common during the cold winter months [34,35] and gastrointestinal infections are more common during the hot summer months [35], the longer, colder winters and shorter, cooler summers in northern Sweden will make the effect of being born during winter on neonatal mortality more pronounced compared to the Mediterranean countries.

Contemporary research has revealed that the combination of low temperatures (below 0 °C) in combination with dry air increases the incidence of several respiratory pathogens [36], and this weather combination is representative of northern Sweden. This weather combination in the north, compared to other areas of Europe where the winter climate is generally warmer and more humid, is an important factor for further research concerning seasonality and infant mortality. Because the exposure to disease varies by seasonality, there are reasons to study the relationship between month/season of birth and causes of death among infants [37]. For example, Reher and Sanz-Gimeno found in their study of the association between month of birth, age at death, and cause of death that the closer a child is to birth and being born during winter, the greater the risk of death due to respiratory infections [11]. As Reher and Sanz-Gimeno conclude, “The worst situation for a young child was to be very young (0–1 months) during the winter season” ([11], p. 120). This was also true for the Sami infants in this study. The association between climate and temperature at birth and death is another area for further comparative research.

Another vital factor for explaining infant mortality in historical populations is breastfeeding practices [24]. Contemporary research on the impact of breastfeeding on causes of death among infants reveals that the protective effect of breastfeeding during the first six months of life is greater against diarrhoea than against deaths due to acute respiratory infections [38]. This, in combination with the waning effect of antibodies after the age of 6 months might contribute to the higher risk of mortality found among summer-born infants entering winter compared to the corresponding risk for the winter-born entering their first summer, as well as the lack of influence of season of birth for younger infants between 29 and 179 days of age.

While a few studies have addressed the impact of weather on total mortality in pre-industrial Sweden, little is known about its impacts on infants. Previous research showed higher winter temperature to be associated with decreased total annual mortality in northern Sweden and that children aged 3 years and older appeared more vulnerable to harsh weather than younger children [15]. Further, climate vulnerability decreased in the course of industrialization and demographic transition [39]. In this paper, infant mortality has been shown to vary by season of birth, but the contribution of weather variations is not fully clear. Possible factors linking climate conditions to infant mortality are the food availability and the mother’s health and nutritional status, before birth and after birth [24]. In rural communities, food supplies were most scare in the spring, especially after poor harvests, causing a diet that was inferior compared to that of other seasons [10,24]. In contemporary research about infant mortality, the month of birth is often used as a proxy for early life conditions such as nutritional status and exposure to infections and temperatures [40]. Also, the lingering effect of season of birth on adult life expectancy (e.g., factors that arise in utero or during early infancy) has been studied, where being born at the end of the harvest season has been associated with better adult health [41,42,43]. The correspondence between birth month and adult mortality (>50 to 80 years) among the Swedish population revealed a higher mortality among individuals born in spring/summer compared to autumn-born individuals [44]. Bengtsson and Lindström’s study showed that in the south of Sweden, the disease load of infectious diseases during infancy had a lasting impact on mortality later in life [37]. Forthcoming research will handle weather and seasonal effects over a longer time period in a subarctic environment, including more parishes than included in this study. Following the two populations into adulthood is another area for further research as well as studying the seasonal effect on the rate of stillbirths.

## 5. Conclusions

The present study has revealed how season of birth affected infant mortality among two historical populations in Sápmi, a subarctic area of Sweden. The results showed that the neonatal mortality among the Sami population was higher during the winter season and lowest during summer. This difference might be due to the varying work intensity of reindeer-herding Sami women during the year, in combination with differences in climate and risks of infectious diseases between the two seasons. Among both populations, infants born during summer had an increased risk of mortality when entering their first winter at an age of 6 months, and this might be explained as a vulnerable period of weaning and the introduction of food that coexisted with a period of harsh climate with a high risk of infectious respiratory diseases. Future analyses will investigate season of birth among historical populations in a larger subarctic area and over a longer time period and will include more climate-related data such as information about temperature and weather.

## Figures and Tables

**Table 1:** Number of births and number of deaths according to month of birth, 1800–1899 ^1^.

Month of Birth	Sami				Non-Sami			
	Births		Deaths		Births		Deaths	
	*N*	%	*N*	%	*N*	%	*N*	%
January	643	10.5	119	10.9	493	9.9	59	9.6
February	603	9.8	107	9.8	456	9.2	53	8.6
March	621	10.1	114	10.5	444	9.0	50	8.1
April	496	8.1	83	7.6	408	8.2	40	6.5
May	471	7.7	84	7.7	420	8.5	58	9.4
June	471	7.7	88	8.1	363	7.3	43	7.0
July	426	6.9	72	6.6	384	7.7	49	8.0
August	399	6.5	65	6.0	409	8.2	58	9.4
September	488	7.9	80	7.3	377	7.6	50	8.1
October	523	8.5	93	8.5	402	8.1	54	8.8
November	484	7.9	92	8.4	377	7.6	52	8.4
December	527	8.6	92	8.4	425	8.6	50	8.1
Total	6.152	100	1.089	100	4.958	100	616	100

^1^ Source: Demographic Data Base, Umeå University.

**Table 2:** Deaths by age groups in North Sápmi, 1800–1899, births and infant mortality rate ^1^.

Age at Death	Sami		Non-Sami	
	*N*	IMR	*N*	IMR
0–7 days	205	33	132	27
8–28 days	271	44	101	20
29–179 days	408	66	240	48
180–365 days	207	34	143	29
1–4 years	458	74	348	70
Births:	6.152		4.958	

^1^ Source: Demographic Data Base, Umeå University. IMR, infant mortality rate; IMR: deaths per 1000 live births.

**Table 3:** Cox regression of infant and child mortality in Sápmi, 1800–1899, presented as relative risks, (exp (B)) ^1^.

Variates	0–7 days	8–28 days	29–179 days	180–365 days	1–4 years
**Sami**					
Gender (Reference Female)					
Male	1.34 *	1.35 *	1.22 *	1.07	1.11
Parity (Reference 1)		*	**		
2–3	0.70	0.66 *	0.84	0.79	1.23
4–5	0.89	0.85	1.00	0.65 *	1.52 **
≤6	1.08	1.02	1.29	1.04	1.49 **
Season of birth (Reference Winter)		*		*	
Spring	0.87	0.94	0.91	1.41	1.06
Summer	0.83	0.60 **	1.01	1.74 ***	0.84
Autumn	0.81	0.90	1.09	1.11	1.07
Region (Reference Jukkasjärvi)					
Jokkmokk	0.85	0.74 *	1.04	1.00	0.82 *
Overall *p*-value	0.100	0.000	0.030	0.030	0.000
**Non-Sami**					
Gender (Reference Female)					
Male	1.40 *	0.91	1.27	1.13	1.45 ***
Parity (Reference 1)	***	*	***		*
2–3	0.47 ***	0.62 **	0.68 *	0.60 *	0.83
4–5	0.50 **	0.42 ***	0.48 ***	0.87	0.78
≤6	0.60 *	0.75	0.91	0.88	1.06
Season of birth					
(Reference Winter)				***	*
Spring	0.67	0.84	0.14	1.36	1.23
Summer	0.81	0.81	0.91	2.33 ***	0.91
Autumn	1.13	0.62	1.39	1.21	1.33 *
Region (Reference Jukkasjärvi)					
Jokkmokk	1.12	0.60 **	0.69 **	0.63 **	0.71 **
Overall *p*-value	0.002	0.018	0.000	0.000	0.000

* Significant at the 5% level; ** significant at the 1% level; *** significant at the 0.1% level. ^1^ Source: Demographic Data Base, Umeå University.
